# Principles Underlying Surgical Decision-Making in Lowest-Instrumented Vertebra Level Selection of Adolescent Idiopathic Scoliosis: From Traditional Landmarks to Emerging Predictive Models—A Narrative Review

**DOI:** 10.3390/jcm15166241

**Published:** 2026-08-12

**Authors:** Yu-Chun Liu, Chih-Hsuan Yu, Hung-Kuan Yen, I-Hsin Chen

**Affiliations:** 1 Department of Orthopedics, Shin Kong Wu Ho-Su Memorial Hospital, Taipei 111, Taiwan; 2 Department of Orthopedics, National Taiwan University College of Medicine, National Taiwan University Hospital, Taipei 100, Taiwan; 3 Graduate Institute of Biomedical Engineering, National Taiwan University, Taipei 106, Taiwan; 4 Department of Chemical Engineering and Biotechnology, Tatung University, Taipei 104, Taiwan

**Keywords:** adolescent idiopathic scoliosis, biomechanics, lowest instrumented vertebra, surgical decision-making, distal adding-on, machine learning, predictive models, narrative review

## Abstract

Background: Adolescent idiopathic scoliosis (AIS) affects approximately 0.5–5.2% of adolescents. Only a minority of curves progress, and surgery is generally reserved for curves exceeding 45–50°. Selection of the lowest instrumented vertebra (LIV) remains contested and directly influences postoperative coronal balance, distal adding-on (DA) and revision risk. Aim: This narrative review synthesizes the biomechanical principles governing LIV selection, compares traditional anatomical landmarks with contemporary prediction formulas and machine learning (ML) tools, and defines what these tools can and cannot currently deliver at the bedside. Methods: A structured but deliberately non-systematic search of PubMed/MEDLINE, EMBASE and the Cochrane Library (January 2000–December 2024) was supplemented by purposive inclusion of seminal pre-2000 work. No PRISMA-compliant screening, risk-of-bias appraisal or quantitative synthesis was undertaken, and the article is therefore reported as a narrative review. Results: Traditional landmarks—end, neutral, stable, last touched (LTV) and last substantially touched vertebra (LSTV)—are compared; fusion at or distal to the LTV/LSTV lowers DA incidence, yet 8–28% of patients still develop DA. Prediction formulas estimate the residual lumbar curve to within approximately 6°, but each was derived within a single surgical team and none nominates a vertebral level. A composite index combining (LIV− Neutral vertebra) and (LIV− Stable vertebra) reported sensitivity 100% and specificity 92–94% in its derivation cohort, without external validation. Hypercorrection of the main thoracic curve (>53%) combined with postoperative LIV tilt < 10° is associated with DA. Machine learning models predict three-dimensional alignment within 5–7°, yet every published model predicts an outcome; none has shown that acting on a model-derived recommendation prevents DA. Conclusions: LIV selection is best framed as a multidimensional judgment integrating coronal balance, vertebral body rotation, sagittal alignment, skeletal maturity, lumbopelvic morphology and non-radiographic factors such as paraspinal muscle quality, bracing and rehabilitation. Prediction models and artificial intelligence remain hypothesis-generating: external validation and prospective outcome trials are prerequisites before they can be considered practice-changing.

## 1. Introduction

### 1.1. Epidemiology and Clinical Significance

Decision-making in adolescent idiopathic scoliosis (AIS) surgery converges on a deceptively simple question: where should the construct stop? Answering it draws simultaneously on epidemiology (which curves progress), on the history of instrumentation (what correction is achievable), on regional and global biomechanics (what the distal junction will tolerate), on factors that never appear on a radiograph (muscle quality, adjunctive bracing, rehabilitation), and increasingly on computational prediction. These strands are usually discussed in isolation. The purpose of the sections that follow is to connect them, and the review is organized around three questions: (i) What biomechanical property does each traditional landmark actually encode, and where does it fail? (ii) Do multidimensional indices and statistical models improve on single-landmark rules, and at what evidentiary cost? and (iii) What would have to be demonstrated before machine learning tools could legitimately change operative practice?

Adolescent idiopathic scoliosis (AIS) represents the most common form of pediatric spinal deformity, affecting approximately 0.5–5.2% of adolescents depending on the population studied and diagnostic criteria employed [1,2]. This three-dimensional (3D) structural deformity of the spine, characterized by lateral curvature > 10° (Cobb angle) in the coronal plane accompanied by vertebral body rotation and sagittal plane alterations, typically manifests during the pubertal growth spurt between ages 10–18 years [3,4]. Most curves remain mild and require only observation. The frequently quoted figure of approximately 10% requiring intervention should be interpreted with care: it applies to the subset of curves that progress beyond 50°, whereas the great majority of curves exceeding 10° are non-progressive, or remain stable below 50°, and never become surgical candidates. Surgical correction is generally considered for curves exceeding 45–50° in growing adolescents, or for documented progression beyond 40° in skeletally immature patients [5,6].

The biomechanical complexity of AIS extends beyond simple lateral curvature, encompassing intricate relationships between vertebral rotation, rib cage deformity, sagittal profile alterations, and compensatory mechanisms throughout the spinal column [7]. These 3D characteristics have profound implications for surgical planning, correction techniques, and long-term outcomes. Understanding the biomechanical principles governing AIS progression and surgical correction remains fundamental to achieving optimal clinical results while minimizing complications.

### 1.2. Evolution of Surgical Approaches

The surgical management of AIS has evolved dramatically over the past five decades, from the Harrington rod distraction systems of the 1960s through Cotrel–Dubousset instrumentation in the 1980s to contemporary all-pedicle screw constructs [8,9]. These advances have enabled greater correction in all three planes, shorter fusion constructs preserving motion segments, and improved long-term outcomes [10]. However, increased correction capacity has paradoxically introduced new biomechanical challenges, including the phenomena of hypercorrection, compensatory curve progression, and junctional complications [11,12].

Selective thoracic fusion (STF), introduced by Moe in 1958 and refined through subsequent classification systems, represents a paradigm exemplifying the importance of biomechanical principles in surgical decision-making [13]. STF capitalizes on the spine’s inherent capacity for spontaneous correction of non-instrumented compensatory curves through balanced forces and growth modulation [14]. Success depends critically on proper patient selection, appropriate choice of fusion levels—particularly the lowest instrumented vertebra (LIV)—and understanding the biomechanical consequences of instrumentation on adjacent segments [15,16].

### 1.3. The Central Challenge: Lowest Instrumented Vertebra Selection

Among the myriad decisions in AIS surgery, selection of the LIV remains particularly challenging and controversial, with direct implications for postoperative complications including distal adding-on (DA), coronal decompensation, and junctional kyphosis [17,18,19]. DA—defined as progressive angulation (>5°) of the segment immediately distal to the instrumented construct or deviation of distal vertebrae (>5 mm) from the central sacral vertical line (CSVL)—occurs in 8–28% of patients and represents a primary indication for revision surgery [17,20]. The biomechanical mechanisms underlying DA are multifactorial, involving residual growth potential in immature spines, inadequate correction of compensatory curves, vertebral rotation at the distal end of constructs, and mechanical stress concentration at the junction between instrumented and non-instrumented vertebrae [21,22,23].

Traditional guidelines for LIV selection have relied on various anatomical landmarks identified on standing posteroanterior radiographs, including the end vertebra (EV), neutral vertebra (NV), stable vertebra (SV), last touched vertebra (LTV), and last substantially touched vertebra (LSTV) [16,24,25]. While these landmarks provide useful frameworks, outcomes vary considerably, and controversy persists regarding which landmark(s) should take precedence in different curve patterns and clinical scenarios [26,27]. Recent evidence suggests that no single landmark reliably predicts optimal outcomes across all patients, necessitating more sophisticated, integrated approaches [28,29].

### 1.4. Determinants Beyond the Radiograph

It should be acknowledged at the outset that the criteria discussed in this review are overwhelmingly radiological, and that radiological criteria capture only part of the clinical problem. Paraspinal muscle quality, fiber-type distribution and fatty infiltration differ between the convexity and the concavity of the curve and plausibly influence the capacity of the unfused lumbar spine to rebalance after selective fusion [30]. Hybrid strategies—for example a short postoperative brace or continued bracing of the non-instrumented compensatory curve until skeletal maturity—are practiced in some centers and alter the mechanical environment of the distal junction, although comparative evidence is scarce [6]. Scoliosis-specific physiotherapy, trunk-muscle conditioning and perioperative rehabilitation modify functional outcomes independently of the fusion level chosen [6]. Bone quality, ligamentous laxity, body habitus, patient and family expectations, and the operating surgeon’s own technique all contribute further. None of these variables appears in any of the landmark rules or prediction formulas summarized below, and their consistent omission is itself an important limitation of the current evidence base—one to which we return in Section 10.

### 1.5. Scope, Aim, and Organization of This Review

This review synthesizes current knowledge regarding biomechanical principles underlying surgical decision-making in AIS, with particular focus on LIV selection strategies, prediction of postoperative outcomes, and emerging computational approaches. We critically examine:

1. Anatomical landmarks and their biomechanical rationale for LIV selection in different curve types.
2. Factors associated with postoperative complications, including da, coronal imbalance, and residual curve progression.
3. Prediction models and formulas for estimating postoperative alignment.
4. Novel integrated approaches combining multiple biomechanical parameters.
5. Machine learning and artificial intelligence applications in surgical planning.
6. Future directions toward personalized, precision spine surgery.

By elucidating the biomechanical foundations of current practice and highlighting emerging technologies, this review aims to provide clinicians and researchers with a comprehensive framework for understanding and optimizing surgical decision-making in AIS.

Aim. The specific aim of this narrative review is to define, for the LIV decision in AIS, (i) the biomechanical content and the failure modes of each traditional anatomical landmark; (ii) the incremental value, and the methodological cost, of multidimensional indices and regression-based prediction formulas; and (iii) the evidentiary threshold that artificial intelligence and machine learning tools must cross before they can be regarded as practice-ready rather than hypothesis-generating. A secondary aim is to distinguish explicitly between prediction of an adverse distal event and prevention of that event—a distinction that is frequently blurred in the current literature.

## 2. Review Methodology

**Figure 1:**
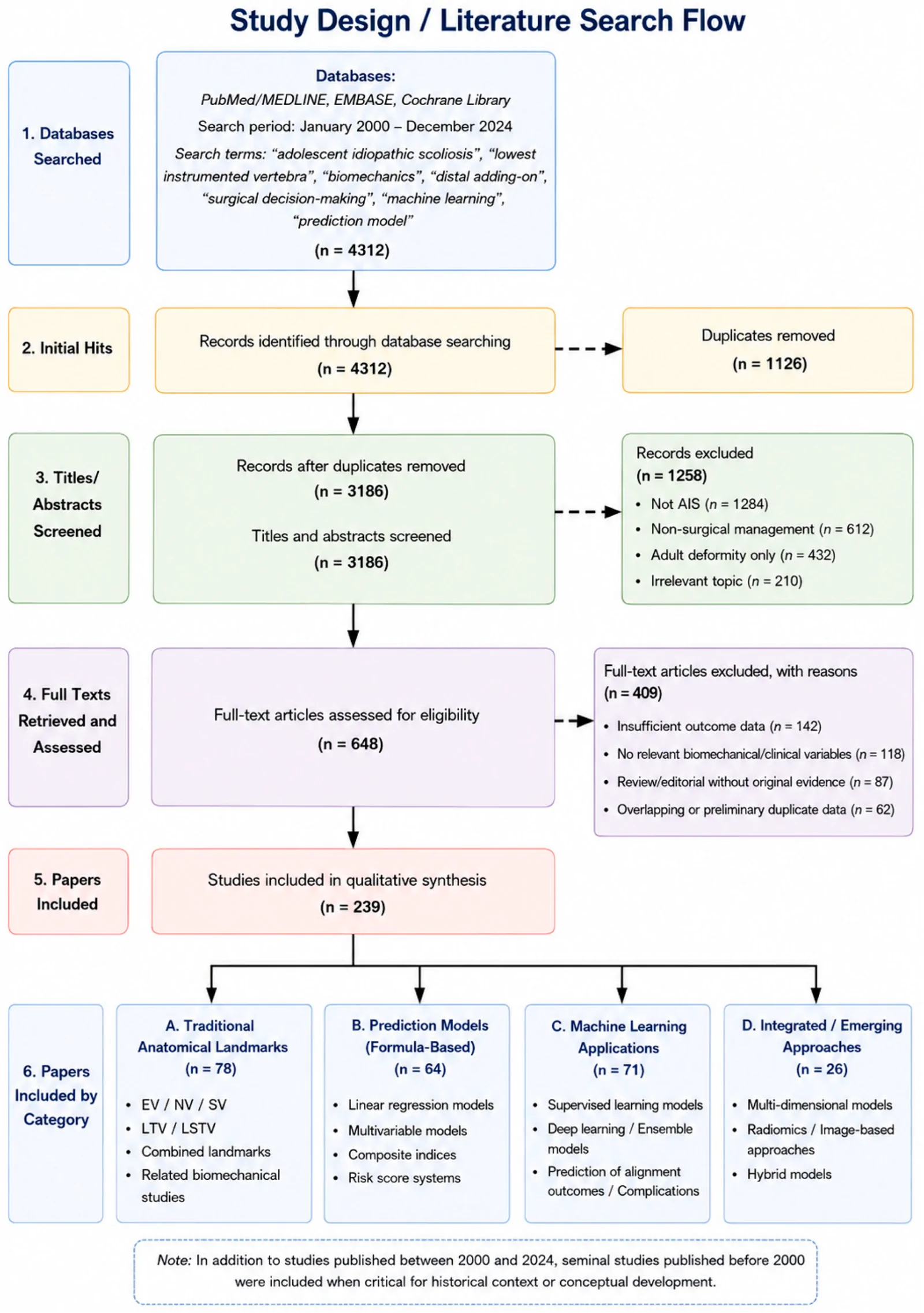
Study design and literature search flow. Records identified in PubMed/MEDLINE, EMBASE and the Cochrane Library (January 2000–December 2024) were screened by title and abstract and then by full text, and the retained studies were grouped into four thematic categories: traditional anatomical landmarks; formula-based prediction models; machine learning applications; and integrated or emerging approaches. Because this is a narrative review, seminal studies published before 2000 were additionally included by purposive selection where required for historical or conceptual context. The diagram is provided for transparency of the search process and is not a PRISMA flow diagram.

This article is a narrative review. It is not a systematic review and is not reported according to PRISMA. A structured search was nonetheless performed to ensure adequate coverage of the field. PubMed/MEDLINE, EMBASE and the Cochrane Library were searched for publications from January 2000 through December 2024. The following Medical Subject Headings (MeSH) and free-text terms were combined using Boolean operators (AND/OR), with no language restriction: “adolescent idiopathic scoliosis”, “lowest instrumented vertebra”, “selective thoracic fusion”, “distal adding-on”, “biomechanics”, “surgical decision-making”, “machine learning”, “artificial intelligence”, “prediction model”, “lumbosacral takeoff angle”, “coronal balance”, “junctional kyphosis” and—added at revision—“pelvis included”, “pelvis excluded” and “pelvic fixation”. Titles and abstracts were screened by two authors, and full texts were retrieved for all potentially eligible records. The flow of records from initial retrieval through to final thematic categorization is presented in Figure 1.

Records were considered eligible if they examined biomechanical factors, anatomical landmarks, prediction formulas or AI/ML applications in AIS surgical planning and reported quantitative radiographic or clinical outcomes; for studies reporting distal adding-on (DA) or junctional kyphosis, a minimum follow-up of 24 months was expected. Records dealing exclusively with non-idiopathic scoliosis, adult spinal deformity or non-operative management were not retained. Consistent with narrative rather than systematic methodology, seminal works published before 2000, relevant reviews, biomechanical simulation studies and cadaveric investigations were additionally incorporated by purposive selection where they provided historical or mechanistic context, and greater weight was deliberately given to recent developments in predictive modeling. Screening was not blinded, no formal risk-of-bias instrument was applied, and no quantitative pooling was performed; the resulting synthesis is interpretive and should be read as such [31]. Regarding the distinction between “pelvis included” and “pelvis excluded” constructs: the pelvis was not a candidate LIV in any retained study, because in AIS the construct terminates within the lumbar spine and pelvic fixation is essentially confined to neuromuscular or syndromic deformity. The distinction nevertheless remains relevant conceptually, since the lumbosacral takeoff angle discussed in Section 5 and Section 6 represents precisely the residual influence of the lumbopelvic junction on a pelvis-excluded construct.

### Organization of the Synthesis

**Figure 2:**
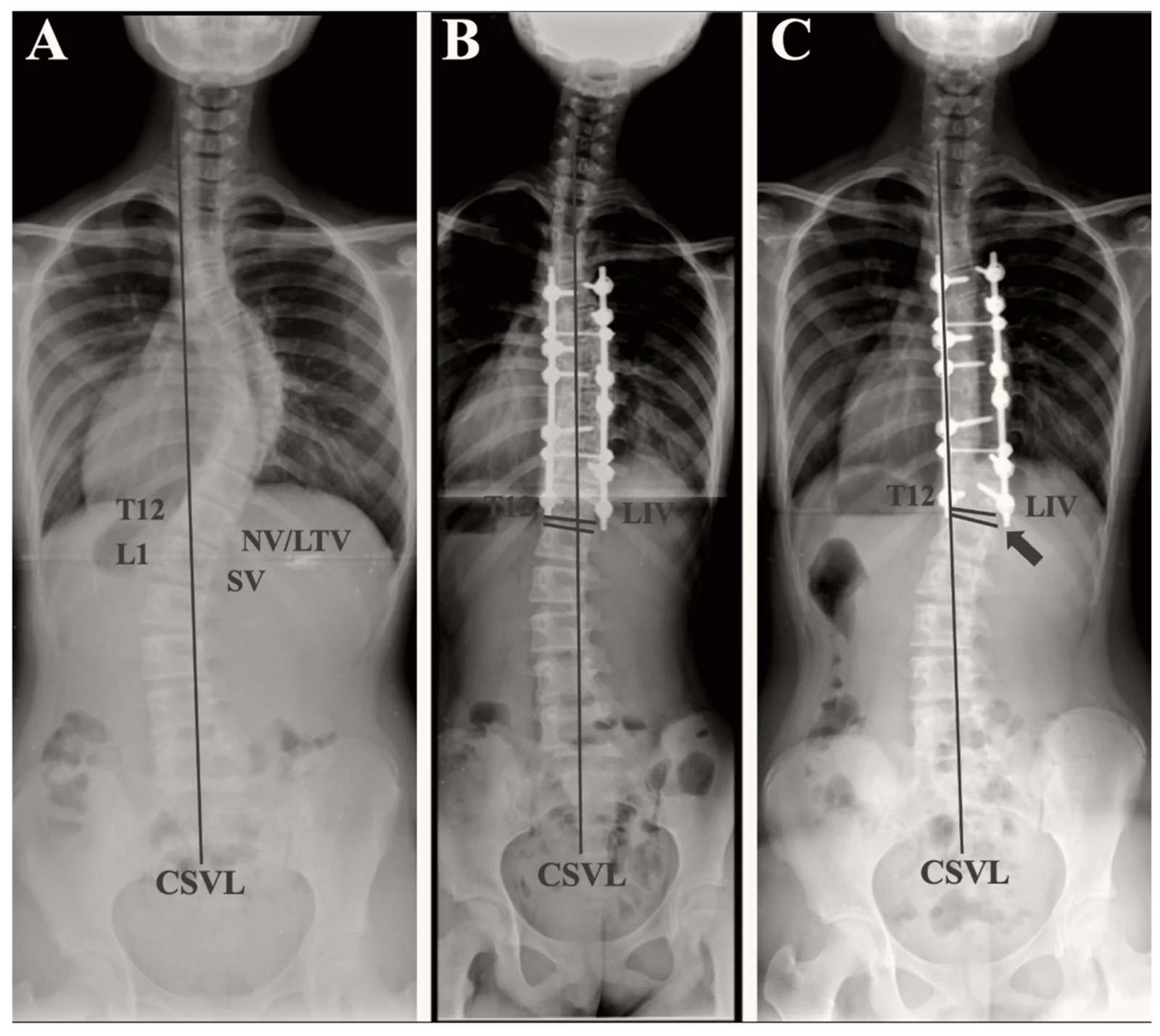
Illustrative case demonstrating landmark discordance, the LIV-index and distal adding-on. (**A**) Preoperative and (**B**) immediate postoperative erect standing whole-spine posteroanterior radiographs of a 13-year-old girl with a Lenke 1BN curve. The LIV, LTV, LSTV and NV all corresponded to T12, whereas the SV lay one level more caudal at L1; the LIV-index for this patient was therefore (LIV − NV) + (LIV − SV) = 0 + (−1) = −1. (**C**) Distal adding-on was evident two years after surgery, as indicated by the arrow. The case illustrates both the discordance between coronal and rotational landmarks discussed in Section 4.3 and the negative LIV-index that flags elevated distal adding-on risk in Section 7. CSVL, central sacral vertical line; LIV, lowest instrumented vertebra; LTV, last touched vertebra; LSTV, last substantially touched vertebra; NV, neutral vertebra; SV, stable vertebra. Reproduced from Chen et al. [90] with permission.

Section 3, Section 4, Section 5, Section 6, Section 7 and Section 8 present the findings of this search and move from mechanism to landmark to model. Section 3 summarizes the biomechanical principles that render the distal junction vulnerable; Section 4 examines the traditional anatomical landmarks, which are compared in Table 1; Section 5 describes the complications that those landmarks are intended to avoid, with an illustrative case in Figure 2; Section 6 reviews regression-based prediction formulas, compared in Table 2; Section 7 addresses composite multidimensional indices; and Section 8 addresses machine learning applications, compared in Table 3. Section 9 considers directions that are plausible but not yet actionable, and Section 10 discusses the synthesis as a whole, including the strengths and limitations of this review.

## 3. Biomechanical Principles of Scoliosis Progression and Correction

### 3.1. Three-Dimensional Nature of Scoliotic Deformity

Contemporary understanding recognizes AIS not as a simple coronal plane curvature but as a complex 3D deformity involving coupled motions in all three anatomical planes [7,32]. In the coronal plane, lateral curvature creates asymmetric loading on vertebral growth plates according to the Hueter–Volkmann principle, whereby increased compressive forces inhibit growth while reduced loading permits continued growth [33,34]. This mechanobiological feedback loop drives progressive deformity during skeletal growth, with the magnitude of asymmetric loading correlating with curve progression risk [35].

Simultaneously, axial plane rotation couples with lateral bending through the biomechanical phenomenon of spinal coupling—where motion in one plane induces motion in others due to the orientation of facet joints and intervertebral disk mechanics [36]. In thoracic scoliosis, vertebral rotation toward the convexity accompanies lateral deviation, creating the characteristic “rib hump” deformity visible on forward bending [37]. Sagittal plane alterations typically manifest as hypokyphosis in thoracic curves, with the normal thoracic kyphosis (T5–T12) often reduced to <20°, significantly affecting both coronal plane mechanics and long-term outcomes [38,39].

### 3.2. Biomechanical Factors in Curve Flexibility and Correction Potential

Preoperative assessment of curve flexibility provides crucial information for surgical planning, with multiple methods employed to evaluate correction potential. Side-bending radiographs, obtained with the patient in lateral decubitus position bending maximally away from the curve convexity, estimate passive flexibility through gravitational and voluntary muscular forces [40]. The flexibility index, calculated as [(standing Cobb—bending Cobb)/standing Cobb] × 100%, typically ranges from 30 to 70% in structural curves, with higher flexibility generally predicting better surgical correction and lower complication rates [41,42].

More recently, fulcrum-bending radiographs, performed with the patient positioned over a padded fulcrum placed at the curve apex, provide information about curve behavior under corrective forces more analogous to intraoperative conditions [43]. Studies demonstrate that fulcrum-bending flexibility better predicts achievable surgical correction compared to side-bending, particularly in less flexible curves where side-bending may overestimate flexibility due to compensatory motion in adjacent segments [44,45].

The mechanical properties of vertebral endplates, intervertebral disks, facet joints, ligaments, and paraspinal muscles collectively determine curve flexibility and correction potential [46,47]. Structural changes including disk wedging, vertebral asymmetry, and facet remodeling accumulate over time, progressively reducing flexibility [48]. Understanding these biomechanical constraints allows surgeons to set realistic correction goals and anticipate the forces required for adequate deformity correction.

### 3.3. Biomechanical Principles of Selective Thoracic Fusion

Selective thoracic fusion exemplifies the application of biomechanical principles to optimize surgical outcomes [49,50]. By instrumenting only the primary structural curve (typically the main thoracic curve), STF preserves motion segments in the compensatory lumbar curve while relying on the spine’s inherent rebalancing capacity [51]. Several biomechanical mechanisms contribute to spontaneous lumbar curve correction following thoracic fusion:

1. Rebalancing of the spinal column as the thoracic curve correction shifts the trunk’s center of gravity, creating corrective forces on the non-instrumented lumbar curve [15].
2. Removal of asymmetric loading allows more symmetric vertebral growth in the unfused lumbar region in skeletally immature patients [52].
3. Ligamentotaxis effects wherein soft tissue tension adjusts to new alignment, contributing to gradual curve improvement [53].
4. Muscular rebalancing as paraspinal muscle forces adapt to the corrected spinal alignment [54].

However, STF success depends critically on proper patient selection and fusion level choice. Excessive correction of the thoracic curve relative to lumbar flexibility may overwhelm compensatory mechanisms, leading to persistent coronal imbalance or lumbar curve progression [11,55]. Conversely, under-correction fails to provide sufficient rebalancing forces, potentially leaving residual deformity and compensatory mechanisms inadequately addressed [56].

### 3.4. Biomechanical Consequences of Instrumentation

Modern pedicle screw constructs provide powerful 3-point (or more accurately, multi-point) fixation enabling substantial deformity correction [57]. However, the mechanical consequences of instrumentation extend beyond the fused segments, creating biomechanical alterations at proximal and distal junctions [58,59]:

#### 3.4.1. Distal Junctional Mechanics

The abrupt transition from rigid instrumented segments to mobile non-instrumented segments creates a stress concentration at the distal junction [60]. Biomechanical studies demonstrate elevated intradiscal pressures, increased facet loading, and altered motion patterns at this transition zone [61,62]. These mechanical factors, combined with growth-related changes in immature spines, predispose to DA and distal junctional kyphosis (DJK) [63].

The orientation of the LIV relative to the CSVL affects these junctional biomechanics substantially. LIV tilted significantly relative to the sacral vertical axis experiences asymmetric loading, with increased compressive forces on the disk’s concave side potentially driving progressive tilt increase and DA [25,64]. Similarly, LIV rotation introduces torsional stresses at the distal junction, contributing to rotational progression of adjacent segments [21].

#### 3.4.2. Proximal Junctional Mechanics

At the proximal junction, similar mechanical factors contribute to proximal junctional kyphosis (PJK), though the mechanisms differ somewhat given the typically greater structural support at upper thoracic levels [65,66]. Studies indicate that abrupt changes in sagittal alignment, particularly fusion ending in the thoracolumbar transitional zone, elevate PJK risk through increased cantilever loading [67].

Understanding these biomechanical consequences informs strategies to minimize junctional complications, including graduated rod contouring, supplemental fixation techniques, and careful attention to sagittal alignment at fusion endpoints [68,69].

## 4. Anatomical Landmarks for Lowest Instrumented Vertebra Selection

### 4.1. Traditional Landmarks and Their Biomechanical Rationale

Multiple anatomical landmarks have been proposed to guide LIV selection, each reflecting different biomechanical considerations. Their definitions, biomechanical rationale, relative advantages, limitations and representative supporting studies are compared in Table 1; the narrative below explains what each landmark encodes and why the landmarks so often disagree.

#### 4.1.1. End Vertebra (EV)

The EV, defined as the most tilted vertebra at the inferior end of the curve, represents the traditional boundary of the structural curve. Biomechanically, the EV experiences maximal lateral wedging and typically demonstrates the most severe disk space narrowing on the curve’s concave side [70]. Historically, King et al. recommended fusion to the lower EV, reasoning that this would address the entire structural curve [71]. However, subsequent experience revealed that stopping at the EV, without considering rotation or CSVL position, frequently resulted in DA, particularly in Lenke 1B and 1C curves [14,15].

#### 4.1.2. Neutral Vertebra (NV)

The NV, defined as the most caudal vertebra demonstrating minimal vertebral body rotation (Nash–Moe grade 0 or I) relative to the sacrum, represents the point where axial plane deformity transitions from structural to compensatory [72]. Suk et al. demonstrated that when the NV is at or distal to the EV, fusion extending to the NV provided satisfactory outcomes with minimal DA risk [73]. The biomechanical rationale rests on minimizing rotational stresses at the distal junction, as instrumenting to a significantly rotated vertebra creates torsional loads predisposing to progressive rotation of adjacent segments [74,75].

#### 4.1.3. Stable Vertebra (SV)

The SV, originally described by King, is defined as the vertebra most closely bisected by the CSVL [71]. Biomechanically, the SV represents the point of optimal coronal balance, where vertebral loading is most symmetric [42]. Takahashi et al., analyzing 172 patients with Lenke 1B, 1C, and 3C curves, found that LIV at or distal to the SV achieved superior main thoracic and compensatory lumbar correction compared to LIV proximal to the SV, with reduced coronal decompensation [76]. The mechanism involves optimizing coronal balance such that residual lumbar curves remain centered over the pelvis, facilitating spontaneous correction through balanced loading [16].

**Table 1:** Traditional anatomical landmarks for LIV selection.

Landmark	Definition	Biomechanical Rationale	Advantages	Limitations	Representative Studies
End Vertebra (EV)	Most caudal vertebra demonstrating maximal coronal tilt within the structural curve	Defines the distal extent of the structural deformity	Simple; historically the earliest landmark; easy to identify	Does not account for vertebral rotation or postoperative coronal balance; associated with higher distal adding-on (DA) rates	King et al. (1983) [71]; Suk et al. (2003) [73]
Neutral Vertebra (NV)	Most caudal vertebra showing minimal or no axial rotation	Minimizes residual rotational imbalance across the distal junction	Better rotational alignment than EV	Identification is subjective; considerable interobserver variability	Suk et al. (2003) [73]; Lenke et al. (2001) [27]
Stable Vertebra (SV)	Vertebra most closely bisected by the central sacral vertical line (CSVL)	Optimizes coronal load transmission through the lumbosacral junction	Better postoperative coronal balance; lower DA risk than EV	Frequently requires longer fusion constructs, sacrificing more motion segments	Harrington (1962) [9]; Takahashi et al. (2011) [76]
Last Touched Vertebra (LTV)	Most proximal vertebra touched by the CSVL	Represents the distal limit of coronal alignment	Shorter fusion than SV while maintaining acceptable coronal balance	Does not consider vertebral rotation or sagittal alignment	Matsumoto et al. (2013) [20]; Cho et al. (2018) [17]
Last Substantially Touched Vertebra (LSTV)	Vertebra in which the CSVL intersects the pedicle or medial pedicle border	Better reflects true distal mechanical stability than LTV	Lowest reported incidence of postoperative DA among traditional landmarks; preserves motion segments	Still based primarily on coronal alignment; does not fully incorporate three-dimensional deformity or patient-specific biomechanics	Qin et al. (2016) [25]; Park et al. (2024) [29]

Abbreviations: LIV, lowest instrumented vertebra; EV, end vertebra; NV, neutral vertebra; SV, stable vertebra; CSVL, central sacral vertical line; LTV, last touched vertebra; LSTV, last substantially touched vertebra; DA, distal adding-on.

#### 4.1.4. Last Touched Vertebra (LTV) and Last Substantially Touched Vertebra (LSTV)

More recent guidelines emphasize the LTV—defined as the most cephalad vertebra in the thoracolumbar/lumbar region touched by the CSVL on standing radiographs—and the LSTV, which requires the CSVL to intersect or pass medial to the vertebral pedicle [25]. These landmarks integrate both rotational (vertebral orientation relative to CSVL) and coronal balance considerations. Multiple studies demonstrate that LIV at or distal to the LTV/LSTV significantly reduces DA incidence compared to more proximal fusion levels [20,77,78].

Cho et al. reported that in 195 Lenke 1A patients, fusion to the LTV resulted in only 2.7% DA incidence compared to 17.6% when stopping proximal to the LTV [17]. The biomechanical explanation posits that LTV represents the caudal extent of significant coronal plane deformity; fusing distal to this point ensures adequate capture of structural deformity while minimizing mechanical stress at the distal junction [79].

### 4.2. Comparative Studies and Meta-Analyses

Several evidence syntheses have attempted to quantify the relative merits of different LIV selection criteria. Liu et al. conducted a meta-analysis of Lenke 1A and 2A curves, examining the relative odds ratios (OR) of DA for various landmarks [28]:

- LIV proximal to SV: OR 4.52 (95% CI 2.56–7.97);
- LIV proximal to EV: OR 3.25 (95% CI 1.84–5.75);
- LIV proximal to NV: OR 2.43 (95% CI 1.66–3.57);
- LIV proximal to LSTV: OR 4.07 (95% CI 1.87–8.89).

These data suggest that all examined landmarks demonstrate statistical associations with DA when violated (i.e., LIV placed proximal to the landmark), with SV and LSTV showing particularly strong associations. However, several important caveats should temper interpretation. The included studies were predominantly retrospective, with heterogeneous definitions of DA, variable follow-up durations (often <5 years), and significant publication bias toward positive findings. Furthermore, the absolute risk remains modest even when guidelines are followed, and conversely, a substantial proportion of patients fare well despite LIV placement proximal to traditional landmarks [15,24].

Park et al. compared nine different LIV selection criteria in Lenke 1A and 1B curves, finding considerable variation in DA incidence (5.9% to 23.5%) depending on the criteria employed. They concluded that criteria incorporating multiple factors (rotation, CSVL position, and end vertebra level) performed best, with the “touched vertebra concept” demonstrating particularly favorable results [29].

### 4.3. Limitations of Single-Landmark Approaches

Despite their utility, reliance on any single anatomical landmark has important limitations:

#### 4.3.1. Variability in Landmark Concordance

Multiple studies document poor to moderate agreement among different landmarks in individual patients [80]. In one study, the NV, SV, and LSTV corresponded to the same vertebral level in only 38% of cases, with discrepancies of 1–2 levels common [76]. This discordance reflects the fact that each landmark captures a different aspect of spinal biomechanics (rotation vs. coronal balance vs. structural curve extent), and these may not align in patients with complex 3D deformities [16]. Figure 2 illustrates the point in a single patient, in whom the NV, LTV and LSTV all lay at T12 while the SV lay one level more caudal at L1.

#### 4.3.2. Two-Dimensional Assessment of Three-Dimensional Deformity

All traditional landmarks are defined using 2D posteroanterior radiographs, potentially missing important 3D characteristics visible on advanced imaging [81,82]. Ohrt-Nissen et al. demonstrated using CT-based 3D reconstruction that vertebral rotation patterns often differ from those inferred from plain radiographs, with implications for LIV selection [83].

#### 4.3.3. Static Assessment of Dynamic Biology

Landmark-based selection typically occurs on a single preoperative radiograph, without accounting for changes during growth, curve flexibility differences, or biomechanical responses to correction [84]. Adolescent spines remain biologically active, with differential growth rates potentially altering optimal fusion level selection over time [85].

#### 4.3.4. Influence of Curve Type and Patient Factors

The optimal landmark varies by curve type, lumbar modifier, skeletal maturity, and other patient-specific factors [27,64]. For instance, LSTV may be most predictive in Lenke 1A curves, while the relationship between NV and SV better predicts outcomes in 1C curves with large lumbar components [76]. This heterogeneity argues against universal application of a single criterion across all patient populations [16].

### 4.4. Contemporary Reviews and Consensus Statements on Level Selection

Four recent syntheses frame the same problem from complementary angles and merit explicit consideration. Seo et al. [86] reviewed optimal LIV selection and concluded that the touched-vertebra concept, rather than the stable vertebra, has become the practical default for Lenke 1A/2A curves, while cautioning that the touched vertebra is itself defined on a single standing coronal film. Lenke et al. [87] restated current concepts in level selection and emphasized that the LIV decision cannot be separated from the choice of upper instrumented vertebra, from curve flexibility, or from the correction magnitude the surgeon intends to achieve—an argument that is consistent with the finding, discussed in Section 5, that the degree of main thoracic correction itself modifies distal junctional risk. Baghdadi and Baldwin [88] reviewed fusion-level selection across curve types and drew attention to the fact that the published rules were derived predominantly in Lenke 1 and 2 patterns and transfer poorly to double and triple major curves. Shah et al. [89] proposed updated criteria incorporating three-dimensional analysis, arguing that coronal landmarks defined on two-dimensional radiographs systematically misestimate the true apical and end levels once vertebral body rotation is taken into account.

These four reviews agree on three points: that no single landmark is sufficient; that the distal junction must be assessed in three planes; and that the underlying evidence remains retrospective. They diverge chiefly on how aggressively the construct should be extended distally, which reflects genuine clinical equipoise rather than contradictory data. The present review is intended to complement them by making the prediction-versus-prevention distinction explicit (Section 10.3) and by presenting the landmarks, formulas and machine learning models in directly comparable tabular form (Table 1, Table 2 and Table 3).

## 5. Factors Associated with Postoperative Complications

### 5.1. Distal Adding-On Phenomenon

Distal adding-on (DA) remains among the most studied complications following AIS surgery, with incidence rates varying from 8 to 28% depending on curve type, LIV selection, and follow-up duration [19,63]. It should be noted that definitions of DA differ across the primary literature, which limits the comparability of these figures. A case previously reported by our group, in which the selected LIV lay one level proximal to the stable vertebra and distal adding-on became evident two years after surgery, is shown in Figure 2 [90]. Recent research has identified multiple biomechanical and patient-specific factors associated with DA risk:

#### 5.1.1. Skeletal Maturity

Multiple studies consistently identify skeletal immaturity as a significant DA risk factor [84,91,92]. Patients with Risser stage 0–2 at surgery demonstrate DA rates 2–3 times higher than those with Risser 4–5 [93]. The biomechanical mechanism relates to continued asymmetric growth at the distal junction, where residual coronal plane tilting or rotation of the LIV creates differential loading on vertebral endplates, driving progressive angulation through the Hueter–Volkmann mechanism [33,34].

Triradiate cartilage status provides another maturity indicator, with open triradiate cartilage associated with substantially elevated complication risks including DA [94]. Sponseller et al. reported that among patients with open triradiate cartilage undergoing fusion, 15% developed DA compared to 3% in those with closed cartilage [19].

#### 5.1.2. LIV Characteristics

Beyond the anatomical landmark considerations discussed previously, specific characteristics of the selected LIV significantly impact DA risk:

*LIV Tilt Angle*

The immediate postoperative LIV tilt angle—measured as the angle between the LIV inferior endplate and a horizontal reference line—is mechanistically significant because it directly governs the coronal loading environment of the first mobile disk below the construct. When the LIV endplate is tilted, the subjacent intervertebral disk experiences asymmetric compressive stress: the concave side is subjected to increased load while the convex side is relatively unloaded. In skeletally immature patients, this asymmetric loading activates the Hueter–Volkmann mechanism at the vertebral growth plates of the adjacent non-instrumented vertebra, promoting differential growth that progressively amplifies tilt and drives DA. In skeletally mature patients, the same loading asymmetry promotes accelerated disk degeneration and facet arthrosis at the distal junction, which may also produce progressive angulation over time. Furthermore, LIV tilt misaligned relative to the LSTA creates a kinematic mismatch that alters the neutral zone mechanics of the entire lumbosacral segment, redistributing load across the unfused lumbar curve. This LIV tilt angle demonstrates strong associations with both DA and residual lumbar curve behavior [17,95]. Chen et al. developed prediction models showing that postoperative LIV tilt was the strongest correlate of residual lumbar curve magnitude (R^2^ = 0.86 for immediate postoperative curves, R^2^ = 0.59 for final follow-up) [55].

Sensitivity analysis revealed that postoperative LIV tilt < 10° predicted DA with 97% sensitivity and 50% specificity (OR 35.0), while the combination of LIV tilt < 10° and main thoracic correction > 53% demonstrated OR 16.3 for DA prediction [55]. The biomechanical interpretation suggests that inadequate LIV tilt creates persistent asymmetric disk loading, particularly when combined with relatively overcorrected thoracic segments that generate increased corrective forces transmitted to the compensatory lumbar curve. Importantly, Cho et al. [17] highlighted an additional multidimensional determinant of DA: a mismatch between the postoperative LIV tilt angle and the lumbosacral takeoff angle (LSTA). When the LIV tilt is reduced below the LSTA—meaning the distal instrumented vertebra is tilted more steeply than the sacral base—the resulting coronal and sagittal misalignment at the distal junction imposes asymmetric compressive loading on the subjacent disk, mechanistically predisposing to progressive angulation and DA. This concept underscores that optimal LIV selection requires not only attention to absolute tilt angle thresholds but also to the harmony between LIV orientation and lumbopelvic alignment—a key argument for multidimensional rather than univariate decision-making [55].

*LIV Rotation*

Several investigations have identified vertebral rotation at the LIV as an independent DA risk factor [21,75]. He et al. found that preoperative LIV rotation > Nash–Moe grade I was associated with a 3.2-fold-increased DA risk in Lenke 1A/2A curves [21]. Qin et al. reported that when LSTV-1 (one level above LSTV) demonstrated greater rotation, choosing it as LIV significantly elevated DA incidence [25].

The biomechanical rationale involves torsional stress concentration at the distal junction when instrumenting to a significantly rotated vertebra. When a rotated vertebra is selected as LIV, the orientation of its endplate relative to the disk below is not purely coronal: the disk is already experiencing a torsional preload due to vertebral malorientation. Derotation maneuvers during surgery reduce axial rotation within the fused construct but create a step-change in rotational alignment at the distal junction, concentrating torsional stress at the first mobile disk. This torsional moment at the distal junction—analogous to a torque applied at the base of the construct—drives progressive axial rotation of the subjacent vertebra through the path of least resistance, initiating DA. Additionally, a rotated LIV repositions the facet joints at the distal junction into an orientation that is less resistant to lateral bending, further reducing stability against coronal plane progression [75].

#### 5.1.3. Degree of Main Thoracic Correction

Emerging evidence suggests that excessive thoracic curve correction relative to lumbar flexibility may contribute to DA and lumbar curve progression. Chang et al. analyzed false double major curves (Lenke 1C) and found that thoracic overcorrection—defined as correction exceeding the thoracic bending flexibility—was associated with progressive lumbar curves and late coronal decompensation [11].

Chen et al. identified main thoracic correction > 53% as a DA risk factor (not simply a risk factor for residual lumbar curve progression, which is a related but conceptually distinct phenomenon), particularly when combined with postoperative LIV tilt < 10° [55]. The proposed mechanism involves a “rebound phenomenon” where excessive thoracic correction overwhelms the lumbar curve’s compensatory capacity. The biomechanical imbalance created by hypercorrected thoracic segments generates forces promoting lumbar curve progression as the spine attempts to restore global balance [12].

### 5.2. Residual Lumbar Curve Progression

Beyond the specific DA phenomenon, progressive worsening of residual unfused lumbar curves represents a broader concern following selective thoracic fusion. Multiple factors contribute to this progression:

#### 5.2.1. Lumbar Curve Characteristics

Preoperative lumbar curve magnitude, flexibility, and structural characteristics influence postoperative behavior. Stiffer lumbar curves (flexibility < 60–70% on bending radiographs) demonstrate reduced spontaneous correction capacity and increased progression risk [56,96]. Lumbar curves with significant structural changes—including apical vertebral wedging > 5°, lateral spondylolisthesis, or disk space narrowing—behave less predictably than more flexible compensatory curves [97].

The lumbosacral takeoff angle (LSTA), measured between the sacral endplate and a horizontal reference, reflects lumbopelvic alignment and significantly influences lumbar curve behavior [98]. Chen et al. incorporated LSTA into their prediction model, finding it contributed significantly to both immediate postoperative (β = 0.598) and final lumbar curve magnitude (β = 0.360) [55]. Higher LSTA (more oblique sacral endplate) correlates with larger residual lumbar curves and reduced correction capacity [98].

#### 5.2.2. Thoracic–Lumbar Relationship

The preoperative relationship between thoracic and lumbar curve magnitudes predicts postoperative lumbar behavior. The main thoracic: thoracolumbar/lumbar (MT:TL/L) Cobb angle ratio provides useful prognostic information, with ratios < 1.2 suggesting major lumbar structural components unlikely to correct spontaneously with selective thoracic fusion [99,100].

Lenke’s lumbar modifier system (A, B, C) categorizes this relationship, with modifier C curves (lumbar curve apex more lateral than thoracic apex relative to CSVL) demonstrating the largest residual lumbar curves and highest progression risk following STF [16,27]. Some surgeons advocate for nonselective fusion in modifier C curves, though debate continues regarding optimal management of these complex patterns [101].

### 5.3. Coronal Decompensation

Coronal decompensation, defined as coronal imbalance > 20 mm (C7 plumb line deviation from CSVL), affects 3–15% of AIS patients postoperatively and significantly impacts patient satisfaction and functional outcomes [102,103]. Risk factors include:

- Preoperative coronal imbalance > 15 mm [103];
- Large structural lumbar curves (>40–45°) [15];
- Lumbar modifier C with incomplete lumbar correction [16];
- Excessive thoracic correction relative to lumbar curve [11];
- LIV selection distal to LSTV without adequate lumbar flexibility [24].

The biomechanical basis involves inadequate postoperative balance between corrected thoracic and residual lumbar curves. When thoracic correction shifts the upper trunk’s center of gravity but lumbar curves fail to correct proportionately, persistent lateral shift results [104]. Alternatively, excessive lumbar correction beyond neutral alignment may overcorrect, creating imbalance toward the opposite side [63].

### 5.4. Distal Junctional Kyphosis

While less common than DA in AIS compared to adult deformity surgery, DJK occurs in 2–8% of adolescent patients and represents another biomechanical complication at the distal junction [18]. Risk factors include:

- Fusion ending in the thoracolumbar junction (T12-L1) [18];
- Preoperative thoracolumbar hyper-kyphosis [105];
- Significant sagittal plane correction > 20° [105];
- Osteopenia or poor bone quality [106];
- Stopping proximal to sagittal stable vertebra [105].

Recent investigations by Wang et al. identified postoperative thoracic kyphosis ≥ 25° and thoracolumbar kyphosis ≥ 10° as significant DJK predictors, with LIV selection above the sagittal stable vertebra showing 13.2% DJK incidence versus 0.8% when at/below the sagittal stable vertebra [105]. These findings highlight the importance of sagittal plane considerations in addition to coronal landmarks for comprehensive LIV selection [107].

## 6. Prediction Models and Formulas for Surgical Outcomes

### 6.1. Early Prediction Models

Attempts to mathematically predict postoperative alignment date to the early 2000s. Dobbs et al. developed one of the first predictive formulas for residual lumbar curve magnitude following selective thoracic fusion in 52 patients [56]:

Predicted postoperative lumbar Cobb = 0.82 × (preoperative lumbar Cobb) − 0.58 × (preoperative thoracic Cobb) + 21.2.

This model achieved moderate correlation (r = 0.60, *p* < 0.001) and introduced the concept that residual lumbar curves could be estimated preoperatively based on curve magnitudes. However, limitations included use of only Cobb angles without considering flexibility, rotation, or other biomechanical factors.

Mason et al. proposed a simpler formula based on the main thoracic: lumbar curve ratio for predicting postoperative lumbar alignment in 73 King type II curves [99]:

Postoperative lumbar Cobb = (Preoperative lumbar Cobb)/(MT:L ratio).

While intuitive, this formula similarly lacked incorporation of flexibility or biomechanical variables beyond curve magnitude.

### 6.2. Contemporary Integrated Models

Recent prediction models have integrated multiple biomechanical variables to improve accuracy. The principal published models, together with their design, input variables, prediction target, reported performance, clinical utility and major limitations, are compared in Table 2.

**Table 2:** Representative prediction models for postoperative alignment and lowest instrumented vertebra (LIV) selection in adolescent idiopathic scoliosis.

Study	Year	Study Design/Sample	Prediction Model	Variables Included	Primary Outcome	Model Performance	Clinical Utility	Major Limitations
Dobbs et al. [56]	2004	Retrospective, *n* = 52	Linear regression	Preoperative thoracic Cobb angle; lumbar Cobb angle	Residual lumbar Cobb angle	r = 0.60	First mathematical model for postoperative lumbar curve prediction	Limited variables; no flexibility or rotational parameters
Mason et al. [99]	1998	Retrospective, *n* = 65	MT:TL/L ratio formula	Thoracic-to-lumbar Cobb ratio	Residual lumbar Cobb angle	Moderate correlation	Simple and clinically intuitive	Ignores flexibility, sagittal alignment, and LIV characteristics
Koller et al. [108]	2019	Retrospective, *n* = 168	Multivariable regression	MT Cobb, lumbar Cobb, lumbar flexibility, lumbar modifier, correction rate	Adequate spontaneous lumbar correction	Accuracy 69%	Demonstrated value of combining multiple biomechanical variables	No external validation
Bachmann et al. [98]	2020	Retrospective, *n* = 140	Regression model	Preoperative lumbar Cobb angle; LSTA	Residual lumbar Cobb angle	MAE 5.9° (R = 0.69)	First model incorporating spinopelvic alignment	Does not consider LIV orientation or surgical variables
Chen et al. [55]	2022	Retrospective, *n* = 130	Multivariable regression	Lumbar Cobb angle; bending Cobb; LSTA; postoperative LIV tilt	Immediate and final lumbar Cobb angle	Immediate: R^2^ = 0.86; Final: R^2^ = 0.59	Introduced LIV tilt as a surgeon-modifiable predictor	Requires postoperative measurements; external validation lacking
Chen et al. (LIV-index) [90]	2026	Multicenter retrospective, *n* = 112	Composite index (LIV − NV) + (LIV − SV)	LIV level, Neutral Vertebra, Stable Vertebra	Distal adding-on	Sensitivity 100%; Specificity 92%	Easy bedside calculation; integrates coronal and rotational alignment	Small event number; single-region validation; evaluates DA rather than global outcome

Abbreviations: LIV, lowest instrumented vertebra; NV, neutral vertebra; SV, stable vertebra; MT, main thoracic; TL/L, thoracolumbar/lumbar; LSTA, lumbosacral takeoff angle; MAE, mean absolute error.

*Koller et al. (2019) Model* [108]

Koller et al. developed both linear regression and binary logistic regression models for predicting lumbar curve correction in 168 Lenke 1 patients. Their model incorporated:

- Preoperative main thoracic Cobb angle;
- Preoperative lumbar Cobb angle;
- Lumbar curve flexibility on bending radiographs;
- Lumbar modifier (A, B, or C);
- Main thoracic correction percentage.

The model achieved 69% accuracy in predicting whether adequate lumbar correction (≤20° residual curve) would be achieved. Interestingly, they found that postoperative main thoracic Cobb angle was the strongest predictor of lumbar curve behavior, supporting the biomechanical concept that thoracic correction magnitude directly influences lumbar curve forces.

*Bachmann et al. (2020) Model* [98]

Bachmann et al. focused specifically on the lumbosacral takeoff angle (LSTA) as a predictive variable in 140 patients:

Postoperative lumbar Cobb = 9.55 + 0.35 × (preoperative lumbar Cobb) + 0.56 × (LSTA)

This model demonstrated R = 0.69 and MAE = 5.9°, performing favorably compared to prior formulas. The inclusion of LSTA reflects appreciation of lumbopelvic biomechanics, as oblique sacral inclination requires larger residual lumbar curves to maintain global coronal balance [109].

*Chen et al. (2022) Model with LIV Tilt* [55]

Chen et al. developed the first prediction model specifically incorporating postoperative LIV tilt angle as an independent variable in 130 patients:

Immediate postoperative lumbar Cobb = −3.54 + 0.102 × (preoperative lumbar Cobb) + 0.0703 × (preoperative lumbar bending Cobb) + 0.598 × (LSTA) + 0.828 × (immediate postoperative LIV tilt).

This model achieved R^2^ = 0.86 (R = 0.93), representing the highest correlation reported to date for immediate postoperative prediction [55]. When predicting final follow-up alignment, the formula simplified:

Final lumbar Cobb = −2.49 + 0.283 × (preoperative lumbar Cobb) + 0.360 × (LSTA) + 0.543 × (immediate postoperative LIV tilt).

Achieving R^2^ = 0.59 (R = 0.77), this model maintained good correlation while acknowledging that long-term outcomes involve additional factors beyond immediate mechanical alignment.

The significance of these models lies in identifying LIV tilt as an *operable* factor—a variable that surgeons can directly control intraoperatively through instrumentation strategy and correction maneuvers. This distinguishes it from patient-intrinsic factors like preoperative curve magnitudes or skeletal maturity that cannot be modified.

### 6.3. Decision Trees and Binary Classification Models

Beyond continuous prediction of residual curve magnitudes, some investigators have developed binary classification models to identify “safe” versus “at-risk” scenarios:

*Pasha and Mac-Thiong (2020) Decision Tree* [110]

Using machine learning decision tree methodology in 83 Lenke 1 patients, Pasha and Mac-Thiong developed classification rules for optimal lumbar curve correction following selective thoracic fusion. Their model identified hierarchical decision points:

1. If Preoperative Lumbar Avt > 40 mm → High Progression Risk;
2. If Lumbar Cobb > 35° and Flexibility < 65% → Moderate-High Risk;
3. If Postoperative Thoracic Cobb < 20° and Lumbar Cobb > 20° → Increased Risk.

The model achieved 78% accuracy in classifying satisfactory versus unsatisfactory lumbar correction outcomes. The decision tree format provides intuitive clinical utility, allowing surgeons to systematically evaluate risk factors during preoperative planning.

*Liu et al. (2020) Meta-Analysis-Derived Guidelines* [28]

Through systematic review and meta-analysis of 1586 patients from 12 studies, Liu et al. quantified the odds ratios for DA associated with different LIV selection criteria. While not a traditional predictive model, these pooled estimates provide evidence-based probabilistic guidance:

- STV/(nSTV + 1) as LIV: 4.6% DA rate (reference);
- LTV as LIV: 8.7% DA rate (OR 1.96);
- LIV proximal to LTV: 23.4% DA rate (OR 6.27);
- LIV proximal to NV: 28.1% DA rate (OR 7.93).

These data enable probability-based counseling about DA risks associated with different fusion level choices [28].

### 6.4. Limitations of Existing Models

Despite substantial advances, current prediction models face several limitations:

*Institution-Specific Derivation and Indirect Relevance to the LIV Decision.*

A limitation that applies to every formula in Table 2, and that is easily overlooked, is that these models were derived within single surgical teams, using that team’s imaging protocol, measurement conventions, implant density and correction philosophy. The regression coefficients therefore encode local practice at least as much as they encode universal biomechanics, and a formula that performs well in its derivation cohort may be systematically biased when applied elsewhere. This is precisely why external validation, rather than internal cross-validation, is the relevant test. A second and more fundamental limitation concerns the prediction target: with the exception of the composite indices discussed in Section 7, these models predict a continuous radiographic outcome—usually the residual lumbar Cobb angle—rather than nominating a vertebral level. They therefore inform the LIV decision only indirectly, by allowing the surgeon to compare the predicted consequences of alternative constructs. None of them outputs an LIV recommendation, and none has been evaluated as a level-selection rule.

#### 6.4.1. External Validation

Most models have been developed and validated within single-institution or limited multi-center cohorts, with minimal external validation in independent populations [111]. Model performance may degrade when applied to different patient populations, surgical techniques, or geographic regions [112].

#### 6.4.2. Incomplete Capture of 3D Biomechanics

Existing models primarily incorporate 2D radiographic measurements, potentially missing important 3D characteristics including true vertebral rotation, rib cage deformity, and sagittal-coronal coupling [81]. Advanced 3D imaging modalities like EOS or CT-based reconstruction might provide additional predictive information [113].

#### 6.4.3. Limited Long-Term Validation

Most models predict immediate postoperative or 2-year outcomes, with minimal data on longer-term (5–10 year) performance [56]. Given that AIS patients face decades of life post-surgery, understanding very long-term biomechanical sequelae remains crucial [114].

#### 6.4.4. Surgeon and Technique Variability

Substantial variability exists in correction techniques, instrumentation density, rod contouring, and derotation maneuvers between surgeons and centers. This “surgeon effect” contributes to outcome variance not captured by patient-intrinsic variables alone [115]. Machine learning studies by Pasha et al. found that “surgeon” ranked among the top predictors of 3D outcomes despite controlling for 20 technical factors, suggesting unmeasured aspects of surgical execution significantly influence results [116].

#### 6.4.5. Static vs. Dynamic Considerations

Current models treat the spine as a static mechanical system at discrete timepoints, whereas the spine is a dynamic, living structure undergoing continued growth and remodeling [94]. Incorporating growth modulation, disc degeneration kinetics, and muscle adaptation over time would enhance long-term predictive accuracy [33].

## 7. Emerging Integrated Approaches: The LIV-Index

### 7.1. Concept and Definition

The observation that NV, SV, and LTV frequently fall at discordant vertebral levels—and that no single landmark reliably predicts optimal outcomes across all patients—has motivated the development of integrated approaches that synthesize multiple biomechanical dimensions into a single decision metric. Chen et al. proposed the LIV-index as one such tool, defined as [90]:

*LIV*-*index* = (*LIV level* − *NV level*) + (*LIV level* − *SV level*)

where vertebral levels are numbered sequentially in the caudal direction. An LIV-index ≥ 0 indicates that the selected LIV lies at or distal to the mean position of the NV and SV, while a negative value signals that the LIV is proximal to this composite reference—the clinically actionable cut-off for elevated DA risk. The formula’s two components encode complementary biomechanical requirements: the (LIV–SV) term captures coronal balance, ensuring the distal junction is positioned near the CSVL and subjected to symmetric disk loading; the (LIV–NV) term captures rotational neutrality, ensuring the distal junction is free of significant axial rotation and its associated torsional stress concentration. By summing these components, the index penalizes configurations in which the LIV is simultaneously too proximal relative to both coronal and rotational criteria—precisely the high-risk scenarios identified across previous single-landmark studies.

### 7.2. Clinical Evidence and Applicability

In a multicenter study of 112 Lenke 1 and 2 patients, all 10 who developed DA had an LIV-index < 0, while no patient with LIV-index ≥ 0 developed DA (sensitivity 100%, specificity 92%, NPV 100%; *p* < 0.0001) [90]. Discriminatory performance was maintained across lumbar modifiers (specificity 84% for modifier A; 95% for modifiers B&C) and was most clinically meaningful in skeletally immature patients under 15 years—the subgroup at highest baseline DA risk—where a positive predictive value of 77% was achieved. The index’s practical utility lies in its simplicity: it can be calculated during routine preoperative planning using standard anteroposterior radiographs without additional imaging or software, and it translates directly into a binary decision rule (LIV-index < 0 → consider extending fusion one level distally). Figure 2 shows a representative patient with an LIV-index of −1 in whom distal adding-on was observed at two years.

Despite these encouraging results, the evidence base warrants cautious interpretation. Although the cohort was assembled across more than one center, all participating centers lay within a single healthcare region and shared broadly similar surgical practice, so the study is best regarded as multicenter in recruitment but single-region in validation. The cohort was modest in size and the number of DA events small (*n* = 10); derivation of the cut-off and assessment of its performance therefore rest on the same data, and overfitting cannot be excluded. Independent external validation in a geographically and technically distinct population has not been reported. The index is also inherently two-dimensional, encoding only coronal and axial plane information; it does not incorporate the sagittal stable vertebra or the lumbosacral takeoff angle, which are increasingly recognized as important determinants of distal junctional behavior. Furthermore, all enrolled patients had the LIV placed at or distal to the LTV, so the performance of the index where this criterion is violated remains unknown. Taken together, the LIV-index represents a clinically meaningful refinement over single-landmark strategies—particularly for flagging high-risk patients who may benefit from extended fusion—but it should currently be used as a supplementary decision-support tool rather than as a standalone criterion, pending prospective multicenter validation.

## 8. Machine Learning and Artificial Intelligence Applications

### 8.1. Overview of AI in Adolescent Idiopathic Scoliosis

The explosion of artificial intelligence (AI) and machine learning (ML) technologies in medicine has reached spine surgery, with numerous applications emerging in AIS diagnosis, classification, outcome prediction, and surgical planning [117]. A 2024 scoping review by Goldman et al. identified 40 studies applying AI to AIS, with 77.5% published in the preceding five years, reflecting rapid growth in this domain [118]. Common ML techniques employed include:

- Convolutional neural networks (CNNs) for image analysis (55%);
- Decision trees and random forests (15%);
- Artificial neural networks (15%);
- Support vector machines, gradient boosting, and other algorithms (15%).

Applications span the AIS care continuum from screening and diagnosis through treatment selection to postoperative outcome prediction [118]. The studies most relevant to surgical planning are summarized in Table 3, which lists for each the algorithm, dataset, input features, prediction target, reported performance, clinical relevance and principal current limitation.

### 8.2. Automatic Radiographic Measurement

One of the most mature AI applications involves automatic measurement of radiographic parameters, addressing the time-intensive nature and inter-observer variability of manual measurements.

#### 8.2.1. Cobb Angle Measurement

Multiple studies have developed CNN-based systems for automatic Cobb angle measurement from standing posteroanterior radiographs [119,120,121]. Performance metrics typically achieve:

- Mean absolute error (MAE): 3–5°;
- Correlation with human raters: r = 0.85–0.95;
- Sensitivity/specificity for detecting surgical-magnitude curves (>45°): 90–95%.

These systems often outperform junior clinicians and approach expert-level accuracy. Some implementations integrate automatic vertebral segmentation, landmark identification, and curve classification in end-to-end pipelines.

#### 8.2.2. Vertebral Rotation Measurement:

Assessing axial vertebral rotation (AVR) traditionally requires either manual application of methods like Perdriolle or Nash–Moe grading—both subject to considerable inter-observer variability—or CT scanning with radiation exposure [122]. ML approaches using deep learning for automated AVR measurement from plain radiographs achieve correlations of r = 0.82–0.88 with CT-based reference standards [123].

#### 8.2.3. Three-Dimensional Reconstruction:

More sophisticated systems attempt 3D reconstruction of the entire spine from biplanar radiographs (e.g., EOS imaging). These provide volumetric models enabling comprehensive assessment of 3D deformity including regional kyphosis/lordosis, vertebral axial rotation, rib cage asymmetry, and spinopelvic parameters [82,113]. While computationally intensive, such approaches may eventually enable detailed patient-specific biomechanical modeling [124].

### 8.3. Curve Progression Prediction

Predicting which patients’ curves will progress remains clinically crucial for treatment decision-making. Traditional predictors include curve magnitude, skeletal maturity (Risser stage, triradiate cartilage status), patient age, and gender [2]. ML approaches attempt to improve prognostic accuracy by integrating multiple variables nonlinearly.

#### 8.3.1. Li and Wong (2024) Systematic Review [125]

A systematic review of 15 studies applying ML to AIS progression prediction found:

- Sample sizes: 38–1780 patients
- Most common algorithms: Random forest, artificial neural networks, logistic regression;
- Common input features: Cobb angle, Risser stage, patient age, curve location, vertebral rotation, curve flexibility;
- Performance: AUROC 0.70–0.91, accuracy 70–88%;
- Most studies focused on prediction at initial visit or 6-month follow-up.

Best-performing models typically achieved 10–15% improvement in accuracy over traditional clinical prediction rules. However, external validation remained limited, with most studies using internal cross-validation or single-center cohorts.

#### 8.3.2. Notable Studies

*Alfraihat et al. (2022)* [126]

Random forest model predicting progression in 495 patients achieved 82% accuracy, identifying Cobb angle at initial visit, Risser stage, and curve location as most important predictive features. Their model outperformed logistic regression and provided interpretable feature importance rankings useful for clinical understanding.

*Noshchenko et al. (2015)* [91]

A gradient boosting model integrating clinical, radiographic, and demographic variables predicted curve progression requiring bracing/surgery with an AUROC of 0.87 in a cohort of 526 patients. Interestingly, parental height and family history contributed significantly to model performance, supporting genetic influences on progression.

### 8.4. Surgical Outcome Prediction

More clinically impactful are ML models predicting postoperative outcomes, enabling evidence-based surgical planning and patient counseling.

*3D Radiographic Outcome Prediction:*

*Pasha and Harms Study Group (2021)* [116]

A landmark study analyzed 212 AIS patients using ML to predict 3D spinal alignment at two-year follow-up following posterior spinal fusion. Input features included:

- 12 preoperative patient-specific parameters (curve magnitudes, vertebral rotation, etc.).
- 8 surgeon-modifiable intraoperative factors (UIV/LIV levels, instrumentation strategy, etc.).

Multiple ML algorithms were compared (random forest, gradient boosting, support vector regression, neural networks), with gradient boosting performing best. The model predicted final coronal Cobb angle with MAE = 6.8°, sagittal kyphosis MAE = 7.2°, and axial rotation MAE = 3.1.

Remarkably, “surgeon” emerged as a significant predictor even after adjusting for 20 quantifiable surgical factors, suggesting unmeasured technical nuances substantially influence outcomes. This finding highlights the challenge of capturing the full complexity of surgical decision-making and execution in computational models.

*Kluck et al. (2021)* [127]

Focusing specifically on spontaneous lumbar curve correction following selective thoracic fusion, Kluck et al. applied ML to 3D reconstructions from 152 patients. Their random forest model predicted final lumbar Cobb angle with MAE = 5.9°, identifying preoperative lumbar flexibility and postoperative thoracic kyphosis as key predictors.

They developed a 3D correction threshold: if postoperative thoracic kyphosis > 20° AND lumbar flexibility > 65%, spontaneous lumbar correction to <25° occurred in >90% of cases. This provides quantitative guidance for patient selection for STF.

### 8.5. Surgical Decision Support

Recent work has progressed beyond outcome prediction toward decision support systems. In a multicenter cohort, Shi et al. developed an AI-enabled planning system predicting both radiographic outcomes and patient-reported outcomes (SRS-22 scores) at 6-month, 1-year and 2-year time points [128]. Their ensemble model, combining gradient boosting, neural networks and random forest, achieved:

- SRS-22 pain domain MAE: 0.42 at 2 years;
- SRS-22 self-image domain MAE: 0.38 at 2 years;
- Coronal Cobb MAE: 5.1° at 2 years.

Importantly, they incorporated explainable AI (XAI) features using SHAP (SHapley Additive exPlanations) values to provide transparency regarding which factors most influenced predictions for individual patients. This interpretability is crucial for clinical adoption, as “black box” models without explanations face resistance from clinicians concerned about understanding model reasoning.

*Complication Prediction:*

Machine learning approaches have also been applied to the prediction of specific perioperative complications. Using a multicenter registry of 6076 AIS operations, Gupta et al. developed models predicting length of stay, estimated blood loss, operative time and complication rates [129]. Their XGBoost models achieved:

- R^2^ = 0.43 for operative time prediction;
- R^2^ = 0.31 for estimated blood loss;
- AUROC = 0.72 for any complication;
- AUROC = 0.78 for neurologic complication.

These models enable risk-stratified benchmarking, where actual outcomes can be compared to ML-predicted expected outcomes adjusted for case complexity. This approach moves beyond crude volume-based or ranking-based benchmarks toward nuanced, risk-adjusted quality assessment.

**Table 3:** Representative machine learning applications in adolescent idiopathic scoliosis relevant to surgical planning.

Study	Year	ML Technique	Dataset	Input Features	Prediction Target	Performance	Clinical Relevance	Current Limitation
Pasha et al. (Harms Study Group) [116]	2021	Gradient boosting	212 patients	Preoperative radiographic parameters; surgical variables	Three-dimensional postoperative alignment	Coronal MAE 6.8°; sagittal MAE 7.2°	Demonstrated feasibility of predicting postoperative spinal alignment	No evidence that ML-guided planning improves clinical outcomes
Kluck et al. [127]	2021	Random forest	152 patients	Lumbar flexibility; thoracic kyphosis; radiographic variables	Spontaneous lumbar curve correction	MAE 5.9°	May assist patient selection for selective thoracic fusion	Single-center retrospective study
Alfraihat et al. [126]	2022	Random forest	495 patients	Cobb angle; Risser stage; curve location	Curve progression	Accuracy 82%	Improved prognostic prediction compared with conventional statistics	External validation unavailable
Gupta et al. [129]	2023	XGBoost	6076 patients	Registry-based perioperative variables	Operative time, blood loss, complications	AUROC 0.72–0.78	Useful for perioperative risk stratification	Does not support LIV selection or intraoperative decision-making
Li & Wong (Systematic Review) [125]	2024	Multiple algorithms	15 studies	Clinical and radiographic variables	Curve progression	AUROC 0.70–0.91	Summarized current evidence supporting ML prediction	Considerable methodological heterogeneity; limited external validation
Goldman et al. (Scoping Review) [118]	2024	Review	40 studies	Multiple applications	Current AI landscape	—	Demonstrated rapid expansion of AI research in AIS	Most studies remain proof-of-concept
Shi et al. [128]	2025	Ensemble learning + Explainable AI (SHAP)	Multicenter cohort	Clinical; radiographic; surgical variables	Radiographic outcomes and SRS-22	Cobb MAE 5.1°; SRS MAE 0.38–0.42	Decision-support platform with interpretable predictions	No prospective trial demonstrating improved patient outcomes

Abbreviations: AI, artificial intelligence; ML, machine learning; MAE, mean absolute error; AUROC, area under the receiver operating characteristic curve; SHAP, SHapley Additive exPlanations; SRS-22, Scoliosis Research Society-22 questionnaire.

### 8.6. Challenges and Limitations in AI Applications

Five constraints currently prevent these tools from entering routine LIV planning, and they are shared across most of the studies in Table 3. First, training datasets are small and heterogeneous: most AIS cohorts contain fewer than a few hundred patients, with variable imaging protocols and measurement conventions between centers [125]. Second, the highest-performing architectures remain difficult to interrogate; explainability methods such as SHAP values partially address this, but clinicians reasonably resist recommendations they cannot audit [118,130]. Third, external validation in independent populations is almost entirely absent, and model performance characteristically degrades outside the derivation cohort [112]. Fourth, regulatory approval, liability, data governance and demonstrable equity of performance across demographic groups remain unresolved [131,132,133], as does integration into existing imaging and record systems [134]. Fifth—and decisive for the present review—no AIS machine learning system has been evaluated prospectively against the outcome that matters. Every model in Table 3 predicts an outcome; none has been tested as an intervention, and no study has shown that a construct selected with model assistance produces fewer distal adding-on events than one selected by an experienced surgeon applying the touched-vertebra rule [135]. Until such a comparison exists, these systems are properly described as prognostic research instruments rather than as planning tools.

## 9. Future Directions Relevant to Level Selection

### 9.1. Patient-Specific Biomechanical Modeling

Patient-specific finite element (FE) modeling aims to simulate the biomechanical response to alternative constructs before operating, by combining three-dimensional reconstruction from CT or biplanar radiography, age-appropriate tissue properties, physiological loading, and explicit modeling of instrumentation at different fusion levels [46,124]. Its direct relevance to the present topic is that it is the only method that can, in principle, compare the predicted distal junctional stress of stopping at one level against the next. Feasibility has been demonstrated—Henao et al. modeled two AIS patients and found higher predicted spinal cord strain in the case complicated by an intraoperative neurological event [136]—but simulation times of hours to days, the need for validation against actual surgical outcomes, and the absence of growth and remodeling from current models keep the approach experimental [137,138].

### 9.2. Intraoperative Technologies

Intraoperative technologies bear on level selection mainly by narrowing the gap between the planned and the executed construct. Navigation and robotic screw placement improve accuracy and may reduce radiation exposure [139]. Instrumented rods with embedded strain gauges could quantify the corrective forces actually transmitted to the distal junction—a variable that is currently invisible to every model discussed above [138,140]. Intraoperative cone-beam CT permits verification of LIV position, screw placement and correction before closure [141]. Whether real-time feedback of this kind would change the level ultimately instrumented, or merely confirm it, is untested.

### 9.3. Patient-Level Phenotyping and Non-Fusion Alternatives

Beyond radiographic morphology, heritability estimates of 40–87% and identified susceptibility loci such as LBX1, GPR126 and POC5 raise the prospect of genetic risk profiling [142,143], while differences in disk flexibility, ligamentous laxity, paraspinal muscle composition and bone quality define biomechanical phenotypes that plausibly explain why patients with identical Cobb angles behave differently [23,30]. Non-fusion growth modulation deserves a more measured appraisal than it sometimes receives. Anterior vertebral body tethering is not a new idea: Crawford and Lenke reported progressive correction by anterior tethering more than fifteen years ago [144], and despite subsequent series describing acceptable two- to five-year results [145,146], growth modulation frequently fails to achieve or to maintain the intended correction, with substantial reported rates of tether breakage, overcorrection and conversion to fusion. Vertebral body tethering therefore does not at present remove the level-selection problem; it relocates it.

### 9.4. Long-Term Outcome Tracking and Collaborative Infrastructure

Finally, the evidence underpinning every rule discussed in this review is short-term. Most outcome studies extend to two to five years, whereas a patient operated in adolescence lives with the construct for decades [114,147]. Registry-based tracking at ten, twenty and thirty years—capturing curve maintenance, adjacent segment degeneration, function and quality of life, and the rate and indication for revision surgery—is the only way to establish whether the level chosen to minimize early distal adding-on is also the level that optimizes lifetime outcome. Infrastructure of this kind is best delivered through collaborative, multidisciplinary deformity networks rather than by individual centers [148,149].

## 10. Discussion

### 10.1. Principal Observations

Three observations emerge from this synthesis. First, the historical progression from end vertebra, to stable vertebra, to touched and substantially touched vertebra is not a sequence of successively better guesses but a sequence of progressively richer biomechanical encodings: the end vertebra encodes the extent of the structural curve, the neutral vertebra encodes rotational neutrality, the stable and touched vertebrae encode coronal load transmission, and composite indices attempt to encode two of these dimensions simultaneously (Table 1). Each rule fails in the plane that it does not represent, which explains why the neutral, stable and substantially touched vertebrae coincide in only a minority of patients. Second, the quantitative models that followed (Table 2) have improved estimation of a radiographic outcome without altering the structure of the decision, because they predict a residual curve rather than nominate a level. Third, machine learning has so far reproduced this same pattern at higher dimensionality (Table 3): better prediction, no demonstrated change in what the surgeon should do.

### 10.2. Comparison with Previous Reviews

Our conclusions align with the recent reviews of Seo et al. [86], Lenke et al. [87], Baghdadi and Baldwin [88] and Shah et al. [89] in rejecting any single-landmark rule and in emphasizing three-dimensional assessment of the distal junction. We differ from them in two respects. Those reviews concentrate principally on which landmark to select; the present review concentrates on what each landmark measures, and therefore on why landmarks disagree—an emphasis that explains, rather than merely reports, the discordance documented in Section 4.3. In addition, previous reviews have generally presented artificial intelligence as an emerging clinical aid; we argue that the current literature does not yet support even that description, for the reasons set out below.

### 10.3. Prediction Is Not Prevention

The most consequential ambiguity in this field is the conflation of prediction with prevention. A model that identifies, with high sensitivity, the patients who will develop distal adding-on establishes an association between a preoperative or immediate postoperative configuration and a later event. It does not establish that altering that configuration alters the event. The two claims require different study designs: the first requires a well-calibrated prognostic model with external validation, whereas the second requires that constructs actually be selected differently and outcomes compared. No published AIS study—landmark-based, formula-based or machine learning-based—has performed the second. This matters practically, because the intuitive corollary of a predictive finding (“the index is negative, therefore extend the fusion”) carries its own cost in sacrificed motion segments, and that trade-off has never been quantified prospectively. Throughout this review we have therefore described these tools as predictive and hypothesis-generating and have deliberately avoided the language of complication reduction.

### 10.4. Strengths and Weaknesses of the Prediction Protocols Reviewed

The protocols summarized in Table 2 and Table 3 share identifiable strengths. They render implicit surgical reasoning explicit and auditable. They quantify risk in a form that is useful for preoperative counseling. Several identify surgeon-modifiable variables—notably the immediate postoperative LIV tilt and the degree of main thoracic correction—which conventional landmark rules ignore entirely. Composite indices in particular are calculable at the bedside from a single standing radiograph, without additional imaging or software. Their weaknesses are equally consistent. All are retrospective. Most are derived from a single institution or a single practice culture, so their coefficients encode local technique. Event numbers are small, particularly for adding-on, which inflates apparent discrimination. Definitions of the outcome vary across studies, so reported incidences are only loosely comparable. Sagittal and true axial parameters are usually absent. And, as argued above, none has been tested as a decision rule. On balance these tools are best used to structure discussion and to flag high-risk patients for closer scrutiny, not to override the judgment of an experienced surgeon.

### 10.5. Strengths and Limitations of This Review

The principal strength of this review is that it brings the anatomical, statistical and computational studies on a single decision into one comparative framework (Table 1, Table 2 and Table 3, Figure 1) and states plainly what each approach does and does not demonstrate. Its limitations are those of its design. It is narrative rather than systematic: study selection was purposive and unblinded, no risk-of-bias instrument was applied, and no quantitative synthesis was attempted, so selection bias in favor of frequently cited and recent work cannot be excluded [31]. The underlying literature is dominated by Lenke 1 and 2 curves treated by posterior all-pedicle-screw constructs, and the conclusions should not be extrapolated to double or triple major patterns, to anterior approaches, or to non-idiopathic deformity. Heterogeneous definitions of distal adding-on across the primary studies limit the comparability of the incidence figures quoted. The comparative tables reproduce study-level summaries as reported by the original authors and were not independently recomputed. Finally, several of the machine learning studies discussed are very recent and have not been replicated, so the appraisal in Section 8 may date quickly.

### 10.6. Remaining Gaps and Research Priorities

Four gaps follow directly from the foregoing. First, external validation: no landmark-derived composite index or prediction formula has been validated in a population independent of its derivation cohort, and this is achievable now using existing multicenter registries. Second, prospective comparison: a study in which the LIV is selected by a defined rule versus by surgeon judgment, with adding-on and patient-reported outcomes as endpoints, is the single investigation that would move the field. Third, sagittal and axial integration: existing composite indices are coronal and axial only, and incorporation of the sagittal stable vertebra and the lumbosacral takeoff angle into a unified index is a tractable next step. Fourth, non-radiographic determinants: paraspinal muscle quality, rehabilitation and adjunctive bracing are absent from every model reviewed here and deserve prospective measurement alongside the radiographic parameters.

## 11. Conclusions

Surgical management of adolescent idiopathic scoliosis has evolved from empiric art towards an increasingly evidence-based discipline through the consistent application of biomechanical principles, the development of predictive models, and the integration of computational technologies. The selection of fusion levels—particularly the lowest instrumented vertebra—exemplifies this evolution, progressing from single anatomical landmarks to multidimensional indices that integrate coronal balance, vertebral body rotation and, increasingly, sagittal alignment.

Emerging approaches like the LIV-index demonstrate promise for improving predictive accuracy by synthesizing multiple biomechanical dimensions into unified metrics; however, the available evidence derives predominantly from single-institution retrospective series, and independent external validation has not yet been reported. Machine learning applications show exploratory potential for advancing surgical planning through pattern recognition in high-dimensional data spaces; yet, as detailed in the preceding sections, the existing AIS ML literature is constrained by small training datasets, absence of prospective validation, incomplete incorporation of biomechanical variables, and limited interpretability. Consequently, current ML tools should be regarded as hypothesis-generating research instruments rather than practice-ready decision aids. Rigorous external validation, algorithmic transparency, and—ultimately—the demonstration of improved patient outcomes through prospective clinical trials are indispensable prerequisites before these technologies can be responsibly implemented in routine surgical planning.

The future of AIS surgery lies in genuinely personalized treatment informed by patient-specific biomechanical modeling, phenotyping and computational decision support. As computational power increases, modeling techniques mature and large-scale outcome data accumulate, these advances may improve individual outcomes—provided that each is held to the standard of demonstrated benefit rather than demonstrated prediction. The ultimate goal is not maximal Cobb angle correction but a biomechanically sound, stable and well-balanced spine that functions optimally throughout a lifetime.

## Data Availability

No new data were created or analyzed in this study.
